# The Question of Shock during Operations under Anæsthesia

**Published:** 1891-02

**Authors:** 


### The Question of Shock During Operations Under Anæsthetics.

A correspondent referring to recent notices of deaths under chloroform, draws attention to the fact that it is expressly stated that in one case deceased was not deeply under when the operation was commenced and completed; whilst in the other, death occurred before the patient was completely anæsthetized. Many observers have noticed what they regarded as reflex syncope during imperfect anæsthesia, and Dr. Lauder Brunton, we believe, both in his lectures and in his "Pharmacology," laid down that imperfect anæsthesia rendered the patient peculiarly liable to cardiac failure through afference of sensory impressions conveyed from cutaneous or visceral nerves. It is certainly a commonly recognized phenomenon that trying the spermatic cord, handling the intestines and dragging upon intra-abdominal viscera produce, even under chloroform, not only marked variations in the pulse, but also variations in the rhythm of respiration. During the experiments made by the recent Hyderabad Commission, it will be remembered that no such interference with the heart's action appeared to occur in dogs, at least as far as the Commission were able to reproduce in the lower animals the more complete conditions of the experiment as presented by human beings undergoing an operation under cholorform. In settling this and kindred questions, we shall probably have to recognize as a factor the difference arising from the greater or lesser elaboration of the nervous system in the animals, human or otherwise, under investigation.—*Lancet.*

CHILBLAINS.—Prof. Morrow is crediting with this apparently excellent formula for chilblains:

R. Acidi carbolici..........................j drachm.
Tincture iodini..........................ij fluid drachms.
Acidi tannici..................... ........j drachm.
Cerat. Simplicis.........................iv ounces.

Misce bene ut ft. ungt. Sig. Apply two or three times a day.

